# Clinical Characteristics and Management of Ocular Metastases

**DOI:** 10.3390/cancers17061041

**Published:** 2025-03-20

**Authors:** Karolina Gerba-Górecka, Bożena Romanowska-Dixon, Izabella Karska-Basta, Ewelina Cieplińska-Kechner, Michał S. Nowak

**Affiliations:** 1 Clinic of Ophthalmology and Ocular Oncology, University Hospital, 38 Kopernika Str., 31-501 Krakow, Poland; romanowskadixonbozena1@gmail.com (B.R.-D.); ewelinacieplinska@gmail.com (E.C.-K.); 2 Department of Ophthalmology, Jagiellonian University Medical College, 38 Kopernika Str., 31-501 Krakow, Poland; 3 Institute of Optics and Optometry, University of Social Science, 121 Gdanska Str., 90-519 Lodz, Poland; michaelnovak@interia.pl; 4 Provisus Eye Clinic, 112 Redzinska Str., 42-209 Czestochowa, Poland

**Keywords:** ocular metastases, ocular tumor, brachytherapy, proton therapy, radiation therapy

## Abstract

Intraocular metastases represent the most common type of intraocular tumors in adults. In most cases, the metastases originate from primary breast and lung cancers. Effective management of patients with intraocular metastatic disease requires a multidisciplinary approach involving ophthalmologists, oncologists, and radiation therapists. The primary goals of treatment are disease control, maintenance of optimal quality of life, and preservation of functional vision. This article provides an in-depth overview of intraocular metastases, with special emphasis on the practical aspects of their diagnosis and treatment based on the most recent literature.

## Simple Summary

This study provides an overview of the clinical and imaging features of intraocular metastases, as well as potential treatment strategies, based on the most recent literature. It focuses on differential diagnosis and emphasizes the challenges associated with accurate diagnosis. It also discusses advancements in radiation and systemic therapies, including novel combinatory approaches that contribute to improved outcomes for these patients. Despite the poor prognosis associated with metastatic disease, management involving collaboration between ophthalmologists, oncologists, and radiation therapists can benefit patients by reducing symptoms, preserving useful visual acuity, and maintaining a relatively good quality of life.

## 1. Introduction

Intraocular metastases are the most common type of intraocular tumors in the adult population. Recently, it was estimated that ocular metastases occur in 8% to 10% of patients with disseminated cancer. Postmortem studies confirm their high prevalence in advanced cancer cases. Moreover, detection rates have increased due to advances in diagnostic tools and improved patient survival [1,2,3].

Most metastases are located in the choroid (88%), iris (9%), and ciliary body (2%), but they can also occur in the vitreous body, retina, and optic disc [1,2]. Due to the rich vascularity of the uveal tract, metastases most often affect the posterior pole of the eye [3]. Intraocular metastases typically present as solitary, unilateral lesions [1,4]. Multifocality is observed in 29% to 35% of cases, while bilateral location—in 21% to 50%. These features can help in the differential diagnosis between metastases and primary tumors, especially melanoma [4,5,6]. Breast cancer is the most common primary malignancy that metastasizes to the uvea, accounting for 39% to 49% of all ocular malignancies [7]. Together with lung cancer, which is the second most common origin, they account for 71% to 90% of all cancers that metastasize to the eye [1,4,5,6,7]. Two-thirds of patients with uveal metastases have a history of primary malignancy [1,4]. In cases of breast cancer, most patients diagnosed with ocular metastases already have a history of this neoplasm. In contrast, in patients with lung cancer, the diagnosis of ocular involvement often precedes the diagnosis of the primary tumor due to its tendency to metastasize early in the course of the disease [1,4]. Other primary sites include the gastrointestinal tract, prostate, kidneys, cervix, thyroid, skin, ovaries, and testes [1]. It is estimated that 85% of ocular metastases coexist with metastatic foci in different locations, occurring synchronously in 4% and metachronically in 75% of cases [8]. Choroidal metastases often coexist with brain metastases [8].

This study aimed to provide a comprehensive and up-to-date overview of intraocular metastases, focusing on their clinical features, diagnostic approaches, and evolving treatment strategies. To ensure a thorough analysis, relevant publications were sourced from medical databases (PubMed and Google Scholar), using key terms related to ocular metastases, including “intraocular metastases”, “choroidal metastases”, and “diagnosis and treatment of uveal metastases”. Totally, sixty-one relevant publications (articles and chapters of books) from years 1993–2024 were identified and included in our analysis. Our review is structured into three main sections: (1) pathogenesis, symptoms, and clinical features; (2) diagnostics; and (3) treatment. Additionally, two separate sections provide an in-depth discussion and summarized conclusions.

## 2. Pathogenesis, Symptoms, and Clinical Features

**Figure 1:**
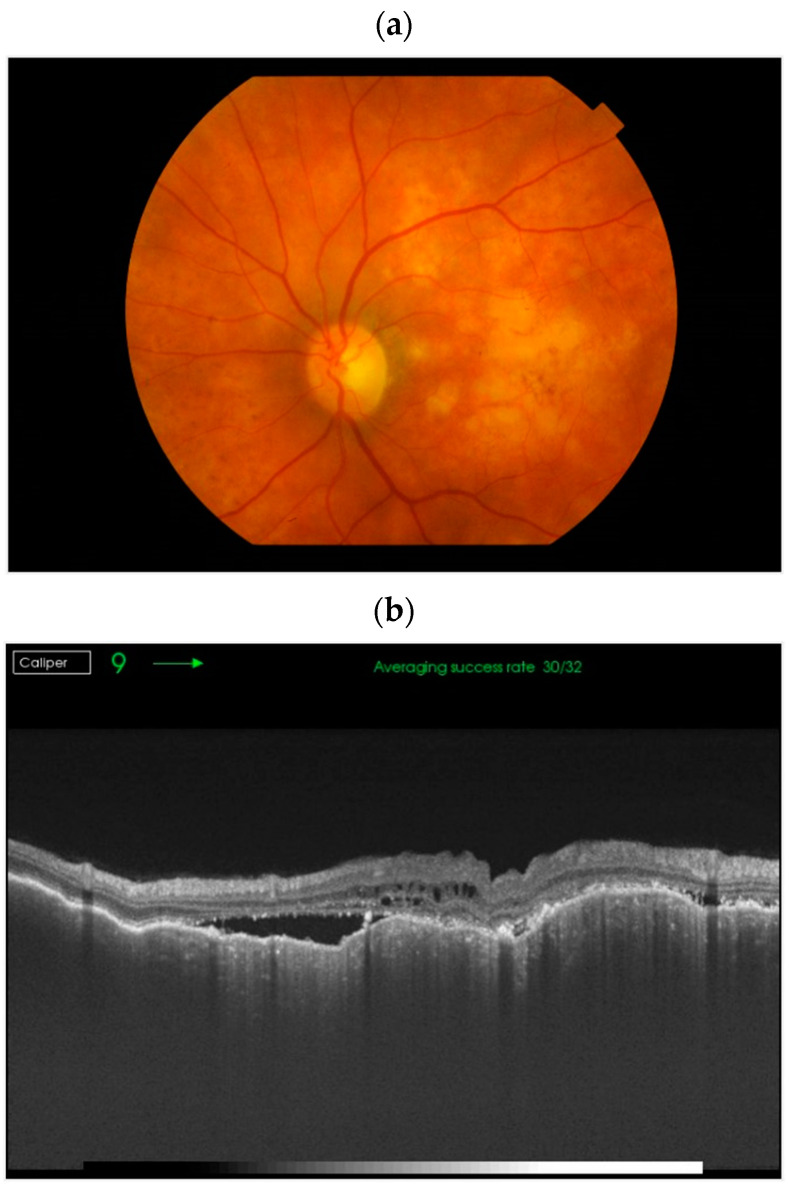
Color fundus photography (**a**) and optical coherence tomography (OCT) scan (**b**) depict the growth of choroidal metastases and their fusion into a diffuse tumor conglomerate with an irregular surface and a complex internal structure associated with subretinal fluid.

Ocular metastases spread hematogenously, with tumor emboli from distant sites lodging in the choroidal vasculature. The distribution of choroidal metastases is determined by the vascularity of the eye [9]. The posterior pole is richly supplied by approximately 20 short posterior ciliary arteries that enter the eyeball near the optic disc and macular region, making it the most common site for uveal metastases [9,10]. This vascular supply explains the common finding of multiple choroidal nodules that merge into an extensive tumor with an irregular, lumpy surface, often coexisting with subretinal fluid (Figure 1) [10]. Metastases to the anterior uvea (ciliary body and iris) reach these areas via the long posterior and anterior ciliary arteries [10].

Although most patients initially report no symptoms, ocular metastases can present with a variety of nonspecific symptoms [5]. The most common clinical manifestation is blurred vision, which occurs in 66% to 98% of patients [5]. Other symptoms include photopsias, metamorphopsias, floaters, pain, and visual field defects, while 13% of patients remain asymptomatic [3]. Visual disturbances are most often caused by macular or peripapillary involvement and the presence of subretinal fluid or exudative retinal detachment [3,5].

**Figure 2:**
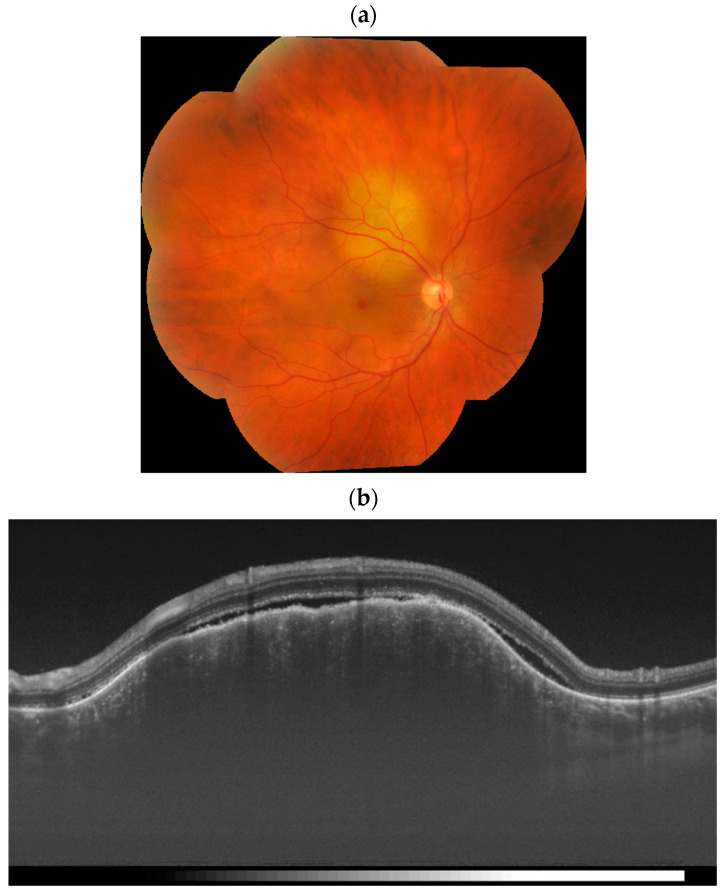
Choroidal metastasis in the right eye of a 65-year-old man with lung cancer, as seen on color fundus photography (**a**) and OCT scan (**b**).

**Figure 3:**
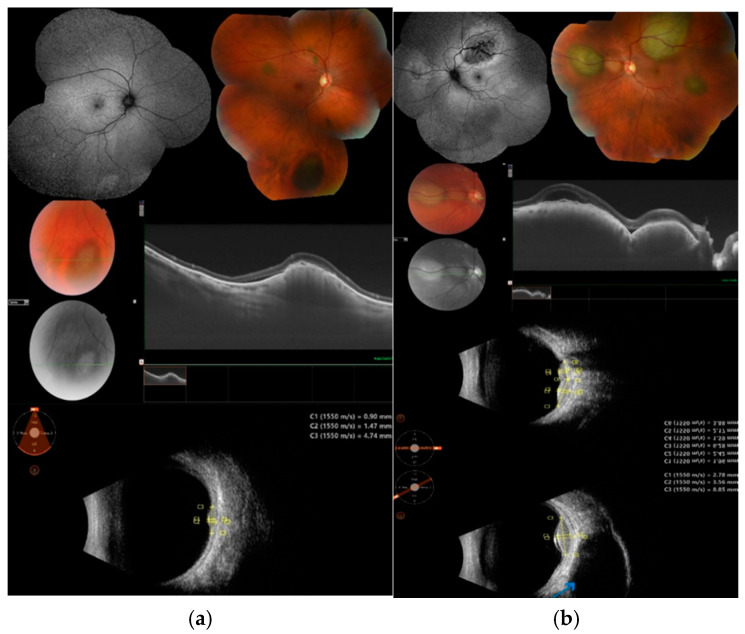
Multiple choroidal metastases in the right (**a**) and left (**b**) eyes of a 32-year-old woman with malignant melanoma of the skin, as seen on fundus autofluorescence, color fundus photography, OCT, and ultrasound. These findings are accompanied by a metastasis to the left orbit, visible on an ultrasound image as a hypoechoic lesion behind the eye wall (blue arrow).

Choroidal metastatic tumors have characteristic morphological features. The lesions are usually located in the posterior pole of the eye. They are creamy white or pale yellow in color, slightly pigmented, with indistinct margins from the surrounding retina (Figure 2) [1,3]. In rare cases, an orange color may indicate a renal, thyroid, or carcinoid origin. Metastases from cutaneous melanoma can be pigmented or amelanotic (Figure 3) [3,11]. Additionally, choroidal metastases may show clusters of dark pigment resembling a “leopard skin” pattern, as well as orange lipofuscin pigment on the tumor surface [3,12,13].

Metastatic foci usually have a large diameter, small thickness, and an irregular surface caused by the merging of small metastatic nodules into a single mass [10]. Typically, choroidal metastases present as flat or plateau-shaped lesions [3,13]. Alternatively, they may be slightly elevated and dome-shaped, often with a multilobular appearance [11,12]. The rupture of the Bruch membrane and the subsequent development of a mushroom shape are rare findings that are statistically more commonly associated with a pulmonary origin of the primary tumor [3,11,14]. Choroidal metastases tend to grow quickly and can spread to other organs [12]. They are often associated with serous retinal detachment (91% of cases) and retinal pigment epithelial changes (around 57% of cases) [4,5].

The differential diagnosis of choroidal metastases includes choroidal osteoma, choroidal hemangioma, posterior scleritis, and amelanotic choroidal melanoma [5,12].

**Figure 4:**
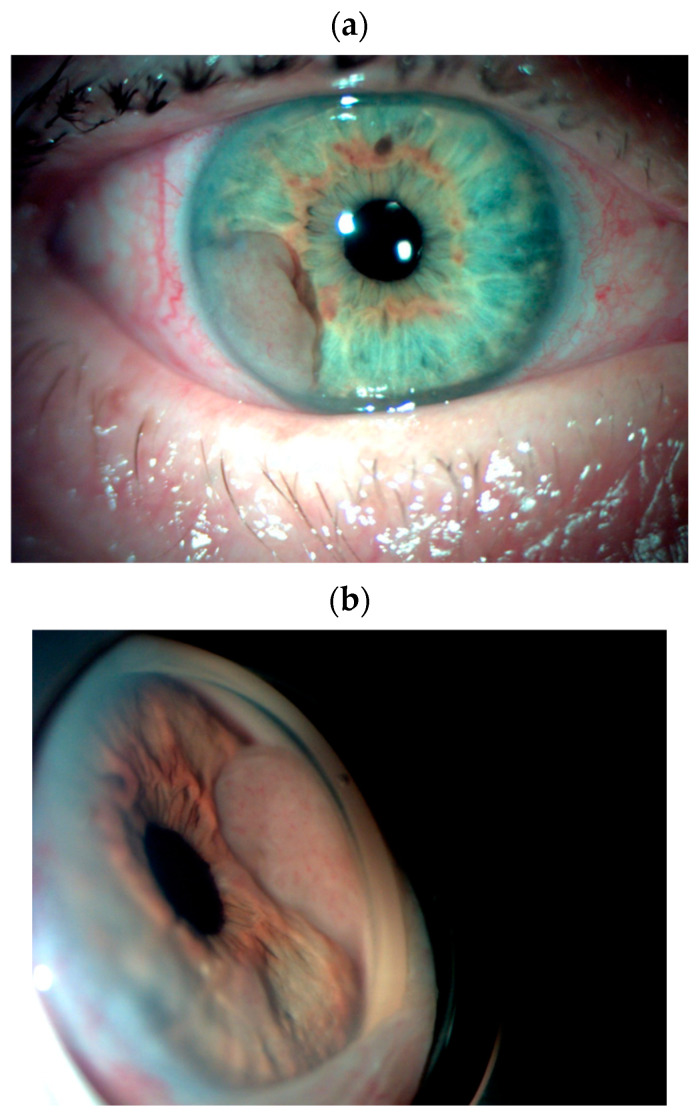

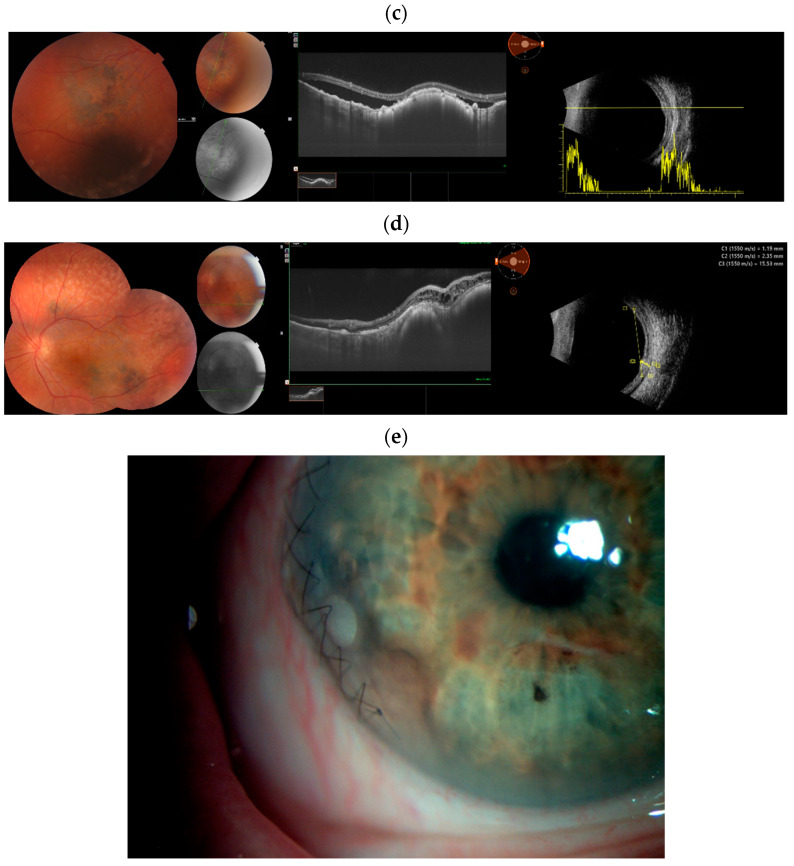
A photograph of the anterior segment of the right eye in a 68-year-old patient with suspected multiple metastatic foci in both eyes. Note the light pink iris tumor in the inferotemporal quadrant ((**a**)—anterior segment, (**b**)—a gonioscopic view of the lesion). Choroidal lesions are visible on color photography, OCT, and ultrasound in the right (**c**) and left (**d**) eyes. The patient underwent extensive investigations, which were inconclusive. However, a biopsy of an excised iris lesion from the right eye ((**e**)—a photograph after biopsy), confirmed by histopathological examination, revealed metastasis from a malignant melanoma. Adjuvant brachytherapy with ruthenium-106 plaque was subsequently performed on the right eye. Despite the presence of ocular metastases, the primary site of the melanoma was not identified. The patient remains under observation in an ocular oncology clinic and has regular follow-up visits in an oncology clinic.

Ciliary body metastases are usually flat, dome-shaped, and amelanotic [3,11]. Iris metastases are often bright white, pink, or yellow and are often found in the inferior quadrant [15]. Other features of iris metastases include anterior uveitis, pseudohypopyon, hyphema, and secondary glaucoma [3,15]. The differential diagnosis of iris metastases includes amelanotic iris melanoma, amelanotic iris nevus, granulomatous iritis, lymphoma, leukemia, and leiomyoma [5,16]. A characteristic feature that distinguishes iris metastases from amelanotic melanoma is the absence of prominent blood vessels within the tumor in metastases (Figure 4) [5,16].

Retinal metastases are rare and present a significant diagnostic challenge, as they can mimic various inflammatory, vascular, and lymphoproliferative diseases. In contrast to choroidal metastases, retinal metastases are typically unilateral and unifocal [3,17,18].

A biopsy may be necessary to establish a diagnosis, especially in complex cases (e.g., an isolated uveal metastasis from an unknown primary site) [3,14]. The identification of the primary malignancy is critical to guide therapy [3]. Biopsy is not a routine procedure and is reserved for patients with no evidence of primary malignancy despite a thorough systemic evaluation [3,5]. For posterior segment lesions, particularly those in the postequatorial region, fine needle biopsy or vitreous cutter biopsy via the pars plana approach is typically used [14]. In contrast, for anterior segment lesions, such as those involving the ciliary body, a transscleral approach may be preferred [3,19].

Usually, the final diagnosis is made based on the patient’s medical history, clinical examination, and additional imaging tests such as ultrasonography and optical coherence tomography (OCT).

## 3. Diagnostics

### 3.1. Ultrasonography

Ultrasonography is used in ophthalmology to localize intraocular masses, estimate their size, and differentiate tissues based on the reflection of sound waves by different tissues (reflectivity). Both B-scan and A-scan ultrasound techniques are employed.

A-scan ultrasonography, a mainstay of current ophthalmological practice, analyzes the amplitude of echoes returning to the transducer. In this modality, the internal acoustic reflectivity of intraocular metastatic tumors is higher than that of most uveal melanomas but not as high as that of choroidal hemangiomas [3,13]. On A-scan ultrasonography, melanoma typically shows high-amplitude initial echoes with low-amplitude internal reflectivity, whereas choroidal metastases are characterized by medium-to-high internal acoustic reflectivity [13].

On B-scan ultrasonography, ocular metastases typically have an irregular structure with an irregular, lumpy surface. They appear as diffuse choroidal infiltrates or dome-shaped lesions, and in rare cases, they may exhibit a mushroom-like morphology [3,12]. Overlying retinal detachment is frequently observed [3]. Ultrasonography is an extremely useful tool for measuring lesions, allowing for the comparison of dimensions on subsequent examinations to assess tumor response to treatment.

Like ocular melanoma, some metastases can develop extraocular extension, which can also be detected using ultrasound [3]. However, a limitation of ultrasonography is its resolution (approximately 50–200 µm), making it challenging to differentiate small tumor entities [13].

### 3.2. Optical Coherence Tomography

**Figure 5:**
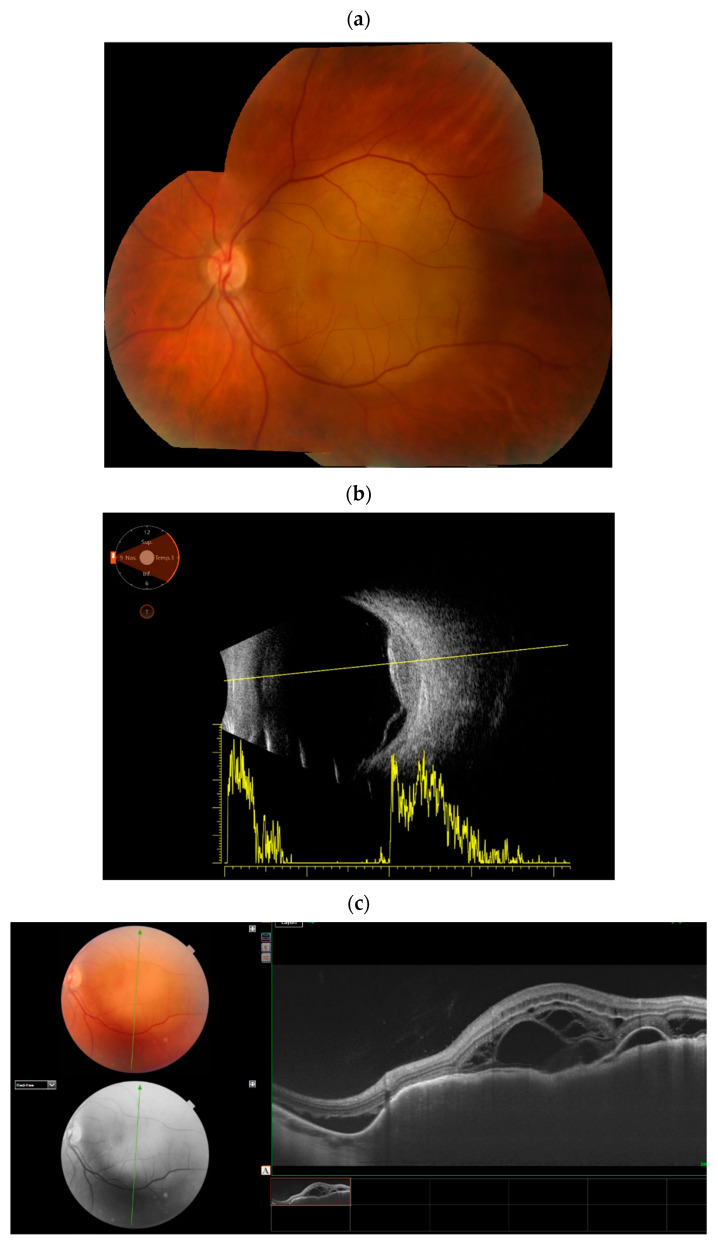
Choroidal metastasis in the left eye of a 37-year-old woman with breast cancer: color fundus photography (**a**) and ultrasound (**b**) showing a lesion with medium internal reflectivity and serous retinal detachment; OCT (**c**) demonstrates an irregular “lumpy bumpy” anterior contour of the tumor, subretinal fluid, and retinal pigment epithelium abnormalities.

Features of metastatic choroidal lesions on OCT include compression of the choriocapillaris, an irregular (“lumpy bumpy”) anterior surface, and frequent presence of subretinal fluid and retinal pigment epithelial changes (Figure 5). Additional features include highly reflective speckles and outer retinal degeneration with photoreceptor layer changes [20].

**Figure 6:**
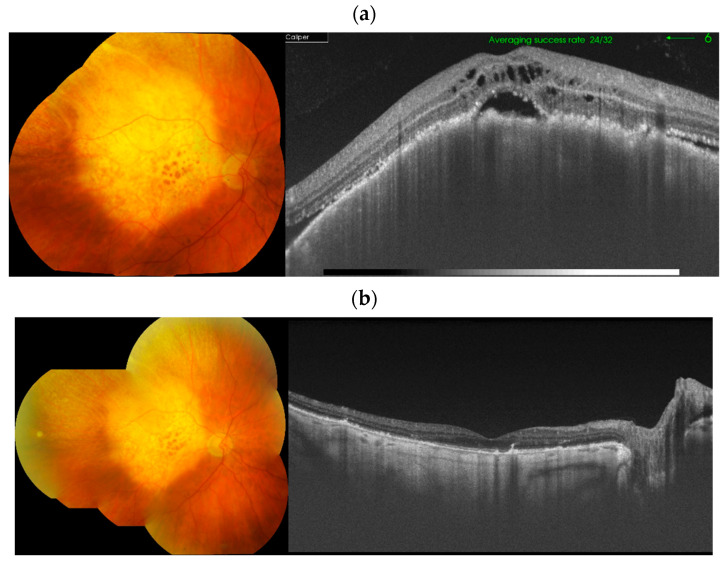
Color fundus photographs and OCT scans presenting choroidal metastases in the right eye of a 55-year-old patient with lung cancer: before treatment (**a**) and after brachytherapy with ruthenium-106, showing a reduction in lesion thickness and complete resorption of subretinal fluid in the macula (**b**).

However, similar to ultrasonography, OCT is useful for monitoring lesions and assessing their response to treatment (Figure 6) [20]. Spectral-domain OCT has a resolution of approximately 5 µm and, according to some authors, is more sensitive than ultrasonography for evaluating small metastatic lesions [13,20]. Notably, OCT angiography allows noninvasive evaluation of choroidal and retinal vascularity. In contrast to choroidal melanoma, hemangioma, and osteoma, which often show a dense vascular network, metastases typically demonstrate no flow at the lesion level and no pathological blood flow in the outer retinal layer [13].

### 3.3. Color Fundus Photography

Color fundus photography is a useful tool for documenting the number and size of tumors and for monitoring treatment [3].

### 3.4. Fundus Autofluorescence

On fundus autofluorescence, ocular metastases typically present with both hyperfluorescent and hypofluorescent foci in areas where the retinal pigment epithelium is disrupted [3]. In addition, hyperfluorescent spots representing lipofuscin pigment may appear. However, these features are not specific to metastatic tumors and can also be seen in other lesions, such as uveal melanoma or hemangioma [3].

### 3.5. Fluorescein Angiography

Fluorescein angiography of metastatic lesions often shows early blockage with late staining and leakage. However, these findings cannot definitively differentiate metastases from other lesions [3]. The double circulation pattern, commonly seen in choroidal melanoma, is not typically present in choroidal metastases [3]. Indocyanine green angiography reveals blockage of choroidal fluorescence and patchy surface staining [3].

### 3.6. Magnetic Resonance Imaging

Although magnetic resonance imaging is not the first-line imaging modality for detecting choroidal metastases, it can visualize extraocular extension and infiltration of intraorbital structures by the tumor. Magnetic resonance imaging may aid in the differential diagnosis between metastases and uveal melanoma, as the latter may show high signal on T1-weighted images [5].

## 4. Treatment

Patients with suspected ocular metastases without a known primary neoplasm should be immediately referred to an oncologist for a thorough evaluation to determine the source of the spread. It was reported that 25% to 34% of patients with lesions of suspected metastatic origin have no previously diagnosed neoplasm, and in 10% of cases, the primary malignancy is not found [1,11]. Thus, ophthalmologists can be the first physicians to evaluate such patients. Patients with a known history of malignancy also require oncological consultation, as the occurrence of new ocular metastases necessitates reassessment of the stage of the underlying disease. The presence of metastases may indicate that the current systemic therapy is ineffective and may necessitate modification.

Each patient with ocular metastases requires an individualized approach to treatment. It is important to note that these patients have generalized cancer, so treatment of ocular metastases is palliative and should be as minimally burdensome as possible. The average survival from diagnosis of choroidal metastases is 9 to 10 months [1,8]. Survival is usually longer in patients with breast cancer metastases and shorter in patients with lung cancer metastases [1,2,4]. Treatment of choroidal metastases depends on the general condition of the patient, the location, size, and laterality of the lesions, and the overall prognosis. Treatment should be aimed not only at prolonging survival, but more importantly, at maintaining an optimal quality of life. Treatment goals also include the preservation of useful vision, preservation of the eye, and reduction of iatrogenic damage to sensitive tissues [3,21]. The management of patients with choroidal metastases requires cooperation between ocular oncology specialists, including ophthalmologists, medical oncologists, and radiation therapists. It should also be tailored to the individual needs of the patient. Systemic therapy is the mainstay of metastatic cancer treatment. Chemotherapy, hormone therapy, and immunotherapy used to treat the primary malignancy are usually effective against metastatic lesions in the eye. These treatment modalities reduce the size of metastatic lesions and are usually sufficient to control them [3,8]. If regression of the lesion is observed, the clinician may opt for regular surveillance without the need for additional local treatment [21]. Sight-threatening uveal metastases may prompt urgent restaging and immediate treatment to avoid vision loss. Painful metastases are also a high priority and usually require rapid intervention [21].

General indications for local treatment of metastatic ocular lesions are presented in Table 1 [5]. Depending on the patient’s life expectancy and response to systemic therapy, local treatment may be implemented. These therapeutic options include surgery, laser therapy, and radiation therapy [3,8].

**Figure 7:**
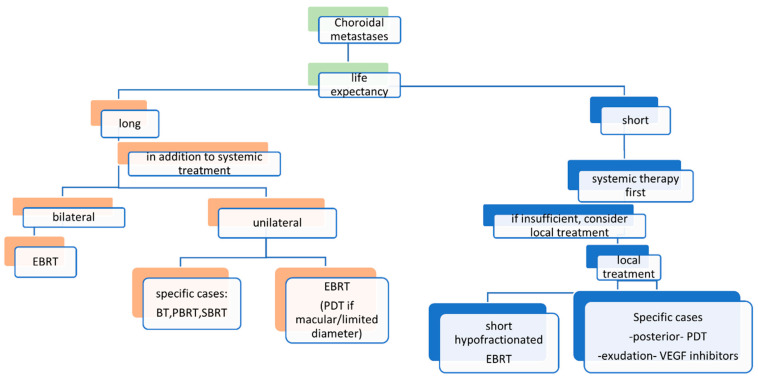
Treatment algorithm for choroidal metastases [21].

A treatment algorithm for choroidal metastases is presented in Figure 7 [21]. For patients with a short life expectancy, simple and quick methods are recommended, including vascular endothelial growth factor (VEGF) inhibitor injections, photodynamic therapy (PDT), and short hypofractionated conventional external beam radiation therapy (EBRT) [21]. On the other hand, for patients with a longer life expectancy, slightly more burdensome but more effective methods are proposed, such as EBRT, brachytherapy, proton beam radiation therapy (PBRT), stereotactic body radiation therapy (SBRT), and PDT (especially in cases with juxtapapillary and macular involvement) [21].

BT, brachytherapy; EBRT, external beam radiation therapy; PBRT, proton beam radiation therapy; PDT, photodynamic therapy; SBRT, stereotactic body radiation therapy

### 4.1. Systemic Therapy

Systemic therapy is administered in patients with disseminated cancer independently of local treatment. While a discussion of all systemic therapy options is beyond the scope of this article, it is important to highlight key strategies used to treat the most common cancers in modern clinical oncology.

**Figure 8:**
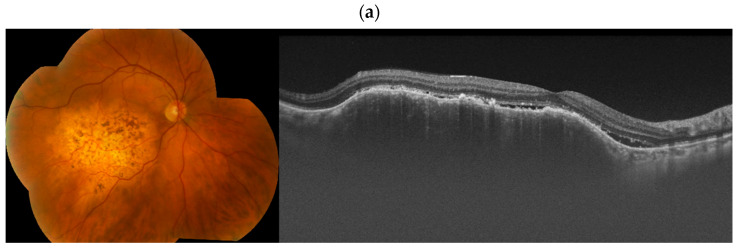

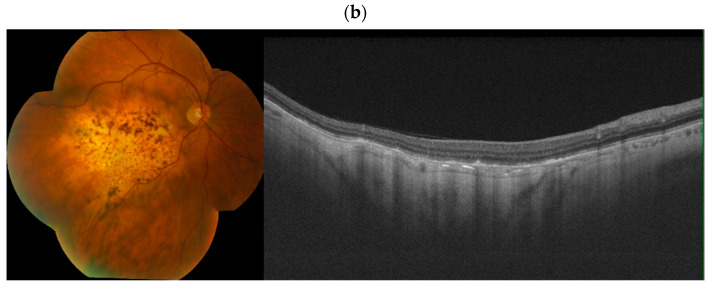
Color fundus photography and OCT of a choroidal metastasis from lung cancer in the right eye of a 66-year-old patient: before (**a**) and after systemic chemotherapy (**b**), showing regression of the choroidal lesion following treatment.

For patients undergoing systemic therapy, a reasonable strategy in eligible patients with no immediate risk of vision loss is to wait and assess ocular changes during treatment. Numerous studies confirmed that choroidal metastases can be significantly reduced or even completely resolved during systemic therapy (Figure 8) [3,22,23,24].

The treatment of patients with breast cancer typically involves a combination of local (surgery, radiation therapy) and systemic (chemotherapy, hormone therapy, targeted biological therapy) options. The choice of treatment strategy depends on numerous factors. The most common polychemotherapy regimen combines alkylating agents with antimetabolites and anthracyclines. The most common hormonal drugs are antiestrogens (e.g., tamoxifen) and aromatase inhibitors (e.g., letrozole, anastrozole) [5]. Biologic therapy is based on targeted therapy using humanized monoclonal antibodies directed against tumor antigens. One example is trastuzumab, which targets the HER2 receptor found on breast cancer cells in approximately 25% of patients [5,25]. Another regimen involves tucatinib, a small-molecule HER2 kinase inhibitor. Unlike trastuzumab, which has limited capacity to penetrate the blood–brain barrier, tucatinib can cross the barrier and thus target central nervous system metastases. This suggests the potential efficacy of certain targeted therapies for ocular metastases [21].

In a large study of 264 patients with ocular metastases from breast cancer, regression of ocular lesions was achieved in 65% of patients receiving chemotherapy and/or hormone therapy [5,6]. Complete regression of choroidal metastases was also reported with newer-generation taxanes [5,26]. Capecitabine (a precursor of 5-fluorouracil) and temozolomide (an alkylating agent with excellent central nervous system penetration) are used in the treatment of patients with central nervous system metastases from breast cancer, suggesting their potential effect on choroidal metastases [5,27,28,29].

The pharmacological treatment of lung cancer is the subject of intense research, with molecularly targeted drugs and immunotherapies being the most promising options. Significant progress has been made in the treatment of advanced non-small cell lung cancer (NSCLC), which accounts for approximately 85% of all primary respiratory cancers. In contrast, the progress has been slower in the treatment of small cell lung cancer and pleural cancer [30]. A wide range of new immunotherapy regimens are effective against NSCLC. The choice of regimen depends on programmed death-ligand 1 (PD-L1) expression and genetic mutations, such as epidermal growth factor receptor, *EGFR*, anaplastic lymphoma kinase, *ALK*, and ROS proto-oncogene 1, receptor tyrosine kinase, *ROS1* [30].

In Poland, on 1 January 2021, osimertinib was introduced as a first-line treatment for patients with *EGFR*-positive non-small cell lung cancer. All recommended treatments are available in the current drug program for patients with common genetic disorders, including activating mutations in the *EGFR* gene and rearrangements of the *ALK* and *ROS1* genes. Patients with PD-L1 expression in 50% of tumor cells may receive immunotherapy alone. In cases with lower PD-L1 expression, immunotherapy (e.g., pembrolizumab) can be combined with chemotherapy [30]. This combination significantly prolongs overall survival while reducing the risk of hematologic adverse events [30]. For small cell lung cancer, which is highly sensitive to chemotherapy, various regimens are used, including combinations of etoposide with cisplatin or cyclophosphamide with doxorubicin and vincristine. However, despite high chemosensitivity, the prognosis of patients with small cell lung cancer remains poor [5,31,32].

### 4.2. Surgical Treatment

Surgical treatment, including enucleation, is reserved for advanced lesions that do not respond to conservative treatment and cause severe eye pain [33].

### 4.3. External Beam Radiation Therapy

EBRT is the treatment of choice in patients with bilateral multifocal lesions or lesion size that precludes effective brachytherapy. The use of EBRT leads to regression of metastases in 94% of patients [3]. The eyeball can be preserved in 98% of patients treated with EBRT, with improvement or stabilization of vision reported in 33% to 73% of cases [34,35]. The standard total dose is approximately 20 to 40 Gy, delivered at fractional doses of 2 to 3 Gy over 2 to 4 weeks [3,21]. In severe cases, a shortened (hypofractionated) regimen may be offered, such as 25 Gy delivered at five fractional doses [3,21]. Tumor response to treatment is assessed 3 months after the completion of treatment [21]. Complications include skin erythema, conjunctivitis, dry eye syndrome, corneal ulcer, iris rubeosis, glaucoma, cataract, radiation retinopathy, and optic neuropathy [5].

### 4.4. Brachytherapy

In the treatment of ocular metastases, applicators with ruthenium-106 (for tumor size < 5 mm) and iodine-125 (for tumor size > 5 mm) are used. Single tumors of less than 18 mm in diameter are eligible for brachytherapy. The method is highly effective, with regression of ocular metastases achieved in more than 90% of cases [8,21]. One of the main advantages of brachytherapy is the shorter duration of treatment compared with EBRT. However, there are some disadvantages, including the need for two minor surgical procedures (suturing and applicator removal) under local anesthesia and sedation and the risk of postradiation complications [3]. Treatment effects are typically assessed approximately 3 months after completion [11,21,36].

### 4.5. Transpupillary Thermotherapy

Transpupillary thermotherapy (TTT) using an 810-nm diode laser was reported to be effective in the treatment of metastatic lesions [33]. This method is particularly useful for small lesions (up to 3 mm in thickness), as it can lead to lesion regression and help maintain useful visual acuity. Some authors described the combination of TTT with brachytherapy or EBRT [37,38]. Potential complications associated with laser use include occlusion of retinal veins or arterial branches, vitreoretinal traction, retinal hemorrhage, retinal neovascularization, and retinal folds [33].

### 4.6. Proton Beam Radiation Therapy

PBRT is not a first-line treatment for ocular metastases [21]. This therapy should be preceded by surgery to place tantalum clips and requires full patient cooperation during treatment, which can be challenging in critically ill patients [21]. PBRT is considered in patients with long-term survival prognosis but with tumors resistant to other treatments [21]. In a study involving 76 eyes, the “light field” technique was used without the need for tantalum markers [5,39]. This technique is better tolerated by patients, with the radiation treatment plan based on the fundus mosaic and ultrasound examination. In this study, regression of ocular lesions was achieved in 84% of patients, and in 98% of cases, the response was comparable to results obtained with EBRT and brachytherapy [33,39]. Complications of PBRT include conjunctival keratinization, corneal ulceration, iris neovascularization, neovascular glaucoma, vitreous hemorrhage, retinopathy, and radiation neuropathy [33].

### 4.7. Stereotactic Body Radiation Therapy

There are limited data on the use of SBRT for the treatment of metastatic lesions in the eyeball. Lesions treated with this technique were too large or too close to the optic disc to be effectively treated with brachytherapy [3,40,41,42,43]. SBRT involves the precise delivery of one or more large doses of radiation (12–25 Gy) to the tumor tissue. Depending on the technology, it can be performed with a CyberKnife or GammaKnife [21]. This method allows for the removal of synchronous cerebral metastases during the same surgical procedure [21]. Although effective, it requires approximately 30 min of continuous fixation during the planning and duration of treatment. Therefore, it cannot be used in patients with significant loss of visual acuity in both eyes. Available eyeball immobilization techniques, including retrobulbar block, are invasive procedures that carry the risk of complications. Since complications should be avoided, especially in patients with metastases, the decision regarding this treatment method should be made individually [21].

### 4.8. Photodynamic Therapy

Photodynamic therapy involves the use of verteporfin, a photosensitizing agent administered intravenously, in combination with diode laser light [21]. Shortly after injection, a 689 nm laser is directed at the fundus lesion to activate verteporfin molecules. This triggers a cascade of reactions that generate reactive oxygen species, inducing a cytotoxic effect and causing thrombosis and necrosis of the targeted tissue [13].

This method is mainly used in ophthalmic oncology for the treatment of circumscribed choroidal hemangiomas, but the global shortage of verteporfin (Visudyne^®^) since 2021 has limited the availability of this treatment [21,33,44].

There are documented cases of PDT being used to treat patients with uveal metastases, with some authors reporting encouraging results for small, solitary choroidal metastases [21,45,46]. Since PDT is relatively easy to perform, it is an optimal choice in palliative care. According to current knowledge, PDT can be considered in patients with small tumors located behind the equator, with a thickness of less than 3 mm and a base diameter of less than 10 mm [21]. Larger tumors, especially those complicated by exudates, can hinder laser penetration and are generally not recommended for this treatment. PDT carries the risk of side effects such as intraretinal or subretinal hemorrhage and choroidal atrophy [45]. Moreover, PDT was also reported to be ineffective in patients with progression of choroidal lesions, particularly in large tumors or multiple metastatic lesions [47].

### 4.9. Intravitreal Vascular Endothelial Growth Factor Inhibitor Therapy

Another local treatment that ophthalmologists can perform independently is the intravitreal injection of a VEGF inhibitor, which reduces angiogenesis by decreasing vessel permeability and growth. The breakdown of the outer blood–retinal barrier caused by a choroidal tumor facilitates drug penetration from the vitreous into the choroid [13].

Studies on a small group of patients showed regression of ocular metastases after intravitreal injections of bevacizumab, a VEGF-A inhibitor approved for use as an intravenous chemotherapeutic agent in certain neoplasms [48,49,50,51,52].

VEGF inhibitors can also be considered as an adjunctive treatment for patients with large tumors complicated by significant exudates, which may not be eligible for PDT [21]. However, the relatively short half-life of these agents and the need for regular patient monitoring should be considered [21].

Some studies reported that VEGF inhibitors are not effective in the treatment of metastatic lesions [53,54]. Currently, VEGF inhibitors are mainly used in the treatment of postradiation complications such as maculopathy and retinopathy. Although they may serve as adjuvant therapy in selected cases, further research is needed to determine their role as primary treatment for metastatic lesions [3].

Recent advances in the field of ophthalmic treatment are promising, especially for patients with limited life expectancy. Unlike radiation therapy techniques, which can take weeks to plan and administer, PDT and intravitreal injections can be performed in a single day, making them accessible to patients with a short life expectancy [21].

## 5. Discussion

Although ocular metastases are the most common type of intraocular tumors in the adult population, there is a scarcity of comprehensive reviews in the literature, and our study fills this gap. By synthetizing findings from a broad range of previous reviews, we sought to minimize potential bias and limitations inherent in individual studies and provide a more comprehensive and balanced perspective, accounting for heterogeneity across different works. This review focuses on differential diagnosis and emphasizes the challenges associated with accurate diagnosis. It also discusses advancements in radiation and systemic therapies, including novel combinatory approaches that contribute to improved outcomes for these patients. In cases of suspected choroidal metastases, it is crucial to distinguish them from various primary intraocular conditions, such as choroidal osteoma, choroidal hemangioma, amelanotic choroidal melanoma, and posterior scleritis.

The key clinical and imaging features that aid in differentiating between primary intraocular tumors are listed in Table 2 [5,12,55,56].

The photos and imaging test results from our own material demonstrate the gradual development of metastatic tumors, which appear as clusters of smaller nodules formed by multiplying cancer cells. The irregular structure and reflectivity of the metastatic tumor in ultrasound and OCT examinations are consistent with the progression of the metastatic mass, as observed in the color fundus photos.

Clinical features facilitate the differential diagnosis by distinguishing not only among intraocular tumors but also between different types of metastases. For example, lung cancer metastases are often unifocal, unilateral, and dome-shaped and exhibit medium-to-low internal reflectivity. In contrast, breast cancer metastases tend to be bilateral, multifocal, and plateau-shaped and have higher internal reflectivity [57]. Metastases from renal and gastrointestinal cancers are typically single, unilateral lesions, while those from thyroid cancer are usually bilateral [57].

Some researchers suggest that the morphology and reflectivity of metastatic lesions can assist in determining the location of the primary tumor [57]. Although identifying the primary tumor based solely on clinical and ultrasound features may be challenging, a combination of findings may be useful in specific cases [57].

Given the substantial burden of metastatic cancer in society and its not uncommon spread to the eye, which can decrease quality of life, screening for ocular metastases in individuals with metastatic disease could be considered. Since access to experienced ophthalmologists is limited, it could be hypothesized that, in the era of technological advancements, Artificial Intelligence (AI)-enhanced screening programs could help identify patients who require further ophthalmologic evaluation.

Recent significant developments in AI tools in ophthalmology have primarily focused on diabetic retinopathy, age-related macular degeneration (AMD), and glaucoma, with applications in prevention, diagnosis, disease prediction, treatment planning, monitoring, and subsequent cyclical evaluation of changes [58]. The implementation of AI in ocular oncology is still in its early stages, with most studies focusing on its application for prognostication in patients with uveal melanoma [59]. Some research has also shown potential for AI in detecting and delineating the boundaries of ocular surface squamous neoplasia (OSSN) and detecting retinoblastoma [60,61]. To the best of the authors’ knowledge, AI has not yet been applied to screening patients with suspected ocular metastases. Since early detection and appropriate treatment can significantly improve quality of life, further advancements in pharmacology and technology may continue to influence treatment strategies.

Managing patients with ocular metastases requires balancing treatment efficacy with the risk of complications and the patient’s overall well-being. Recent advances in systemic therapy have led to increased survival rates in metastatic patients. These individuals may benefit from effective, long-lasting ocular treatments that preserve vision during extended survival [13]. For patients with a limited life expectancy, prioritizing quality of life over the possibility of remission is essential, necessitating conservative treatments that preserve vision. In such cases, emerging “office-based” local therapies (i.e., PDT and VEGF inhibitor injections), which can be performed directly by ophthalmologists and require fewer visits, are crucial. These therapies have the potential to maintain visual function while minimizing side effects and reducing damage to other parts of the eye caused by more aggressive treatments [13].

In summary, advancements in the field of oncology, including improved diagnostic tools, better treatment options, and increased awareness of the prevalence of ocular metastases, have led to a greater number of patients with disease requiring ophthalmologic assessment. Despite the poor prognosis associated with metastatic disease, management involving collaboration between ophthalmologists, oncologists, and radiation therapists can benefit patients by reducing symptoms, preserving useful visual acuity, and maintaining a relatively good quality of life. It is crucial to tailor treatment options to each patient individually, taking into account their overall health and comorbidities.

## 6. Conclusions

The key takeaway messages from this article are as follows:

1. Advanced diagnostic tools, including A-scan and B-scan ultrasound, as well as OCT, enable earlier detection and prompt initiation of appropriate treatment for ocular metastases.
2. The morphology and growth pattern of metastases (clusters of nodules gradually developing into a larger mass) enhance our understanding of the mechanisms underlying metastatic development and help differentiate them from other primary ocular tumors.
3. The primary treatment goals for patients with ocular metastases are disease control, maintaining optimal quality of life, and preserving functional vision.
4. Less invasive, office-based treatments in ophthalmology, such as PTD and intravitreal VEGF inhibitor injections, may help preserve vision while reducing the time palliative patients spend in medical facilities.
5. Novel therapeutic approaches in oncology, including targeted therapies (e.g., trastuzumab, tucatinib) and immunotherapies (e.g., osimertinib and pembrolizumab), show promise in disease control and prolonged patient survival.

## Figures and Tables

**Table 1:** General indications for local treatment of uveal metastases [5].

General Indications for Local Treatment of Uveal Metastases
Vision-threatening lesions: lesions close to the optic nerve or macula with signs of disease activity
Lesion enlargement despite systemic chemotherapy
Painful eye

**Table 2:** Differential diagnosis of choroidal metastases—comparison of clinical and imaging features.

Lesion Type	Choroidal Metastasis	Choroidal Melanoma	Choroidal Osteoma	Choroidal Hemangioma
Clinical appearance	Uni-/bilateral; posterior pole; flat/plateau-shaped; pale yellow; multilobular appearance	Unilateral; pigmented tumor; color can vary from amelanotic to dark brown	Unilateral; posterior pole; orange-yellow lesion with distinct geographic borders	Unilateral; red-orange ill-defined disc shape; choroidal peripapillary or macular tumor
USG (A and B presentation)	Moderate to high internal reflectivity; polygonal or dome-shaped with RD; diffuse choridal thickening; minimal or absent internal vascularity	Initial spike followed by low to moderate internal reflectivity; vascular pulsations seen as spikes; dome-like shape; mushroom shape; choroidal excavation; internal vascularity	High intensity echo spike; dense at higher and lower sensitivities; shadowing behind the lesion (`pseudo-optic nerve`)	High internal reflectivity, broad-based echo spikes; fusiform, biconvex cross-sectional shape; serous RD may be observed
OCT	Dome-shaped elevation of thickened RPE and retina, compression of the choriocapillaris; an irregular (“lumpy bumpy”) anterior surface; SRF; retinal pigment epithelial changes	Serous RD around and overlying the tumor; intraretinal cysts in the overlying retina, loss of retinal architecture overlying the tumor	Latticework reflective pattern; hypo-, iso-, or hyper-reflective photoreceptor loss over decalcified areas	Smooth, gradually sloping anterior tumor interface; vertical expansion of the choroid in the area of tumor with no compression of the choriocapillaris; expansion of the medium and large choroidal vessels; SRF; retinal oedema; photoreceptor loss
FA	Hypofluorescence on the arterial phase and progressive hyperfluorescence during subsequent phases, persistent pinpoint leakage throughout the angiogram	No pinpoint leakage; blockage of background fluorescence; patchy pattern of early hyperfluorescence and late intense staining; double circulation pattern	Early patchy hyperfluorescent choroidal filling with late diffuse staining	Early vascular fluorescence filling, later fast, diffuse fluorescent staining
MRI/CT	Choroidal tumor with intense focus of FDG activity on PET/CT	Contrast enhancement on CT; hyperintense on T1w MRI images	Density as bone on CT scan; hyperintense on T1w images, hypointense on T2w images	Hyperintense on T1w images, isointense on T2w images

RD—retinal detachment, SRF—subretinal fluid, RPE—retinal pigment epithelium, FDG—fluorodeoxyglucose, PET—positron emission tomography, CT—computer tomography, MRI—magnetic resonance imaging, FA—fluorescent angiography, OCT—optical coherence tomography, USG—ultrasonography.
